# Point-of-care C-reactive protein testing in general practice out-of-hours services: tool or trap?

**DOI:** 10.3399/BJGP.2025.0302

**Published:** 2025-08-25

**Authors:** Rebecca Payne, Sarah Mills, Clare Wilkinson, Malene Plejdrup Hansen

**Affiliations:** 1 North Wales Centre for Primary Care Research, North Wales Medical School, Bangor University, Bangor, UK; 2 Nuffield Department of Primary Care Health Sciences, University of Oxford, Oxford, UK; 3 GP Out of Hours, Betsi Cadwaladr University Health Board, Bangor, UK; 4 School of Medicine, University of St Andrews, St Andrews, UK; 5 Center for General Practice at Aalborg University, Aalborg, Denmark

Addressing the overuse of antibiotics is an international priority. Antibiotic use is a key driver of antimicrobial resistance (AMR), estimated to account for 4.95 million deaths per year globally.^1^ Much of the UK primary care prescribing of antibiotics happens within general practice out-of-hours services (defined as unscheduled, urgent primary care, occurring outwith normal office hours). In this setting a high proportion of consultations result in an antibiotic prescription, with 59% of these being prescribed for respiratory tract infections.^2^

This high use of antibiotics in GP out-of-hours is unsurprising but concerning. Consultations in GP out-of-hours often involve high-risk decision-making; patients have a higher prevalence of acute illness, and clinicians have limited access to medical information, laboratory results, or prior knowledge of the patient.^3,4^ Patients often travel long distances for care,^5^ and may attend appointments with expectations of antibiotic prescribing.^6^

*“CRP-POCT … cannot and must not be used to replace careful history taking, careful physical examination, and clinical reasoning.”*

One way to address this problem may be through the use of point-of-care testing (POCT). POCTs have the potential to support prescribing decisions in GP out-of-hours care and to safely optimise antimicrobial usage. POCTs are performed on-site and provide results quickly enough to support decisions made during the consultation. They usually give a clinically useful result in numerical form and circumvent the delays associated with laboratory testing.^3,7,8^ The use of C-reactive protein (CRP)-POCT in primary care patients with acute respiratory tract infections has been shown to reduce antibiotic treatment,^9^ meaning CRP can be used as a biomarker in such patients to support a decision to prescribe or safely withhold antimicrobials. CRP-POCTs thus may have the potential to improve clinical outcomes by improving prescribing accuracy, reducing unnecessary referrals, maximising the effectiveness of care, and reducing costs.^10^ While CRP-POCTs are already recommended in national guidelines for respiratory tract infections (including in primary care settings), and access to CRP testing in urgent care services is part of the NHS England commissioning specifications,^11^ usage in UK GP out-of-hours is rare. Furthermore, effectiveness and cost-effectiveness in primary care settings, particularly GP out-of-hours, have not been studied.

Experience from elsewhere in Europe shows that the use of CRP-POCT needs to be judicious. For example, usage has increased significantly in Danish general practice over the past decades,^12^ including in out of hours services; CRP-POCT use increased from 0.2% in 2003 to 18.3% of all consultations in 2018.^13^ However, rather than supporting a reduction in antibiotic prescribing, a recently published study from Denmark has shown that practices with high CRP-POCT usage prescribe more antibiotics than those who use the test less frequently.^14^ How is it that a test that can be used to identify those patients who don’t need antibiotics, results in more patients receiving them? It may be that the CRP-POCT is now being used too often in Danish general practice, perhaps encouraged by reimbursement policies that pay practices a fee each time the test is performed.^15^ Some Danish general practices perform POCT, such as Strep-A and CRP tests, before the patient has received a clinical assessment.^16^ Such practices mean spurious results in patients with a low pre-test probability of bacterial infection can influence decision making, leading to inappropriate prescribing.

Indiscriminate application of CRP-POCT testing therefore cannot be used to address the challenges of antibiotic overprescribing out of hours. As HL Mencken famously said: *‘For every complex problem, there is an answer that is clear, simple and wrong*‘. Helpful as CRP-POCT may seem to be in the out-of-hours context, it cannot and must not be used to replace careful history taking, careful physical examination, and clinical reasoning. If financial incentives are introduced to drive uptake, these need to be implemented thoughtfully and with robust evaluation of potentially unintended consequences. Appropriate and thoughtful selection of patients is key to generating usable results, with NICE recommending CRP-POCT for patients with a respiratory tract infection only when it is unclear whether antibiotics are needed.^17^ With GP out-of-hours under unsustainable increasing pressure, due to rising demand and increasing medical complexity, time to think is often lacking, and prescribing antibiotics can be a quick solution to getting a satisfied patient out of the consulting room. Any proposed solutions to address out-of-hours antibiotics prescribing will need to take into account the reality of working in busy and pressured urgent care services.

GP out-of-hours is an under-researched setting with wide variations in practice.^18^ The sector would benefit from studies leading to clearer policy and agreed national guidelines on implementation of CRP testing to achieve better risk-targeted care and improve antimicrobial stewardship. Real-world trials of CRP-POCT testing are needed, but research and guidelines alone are not enough to ensure best practice — adequate resourcing is also required to create an environment where best practice is possible.
